# Small Molecule Inhibitors Targeting Gα_i_2 Protein Attenuate Migration of Cancer Cells

**DOI:** 10.3390/cancers12061631

**Published:** 2020-06-19

**Authors:** Silvia Caggia, Subhasish Tapadar, Bocheng Wu, Smrruthi V. Venugopal, Autumn S. Garrett, Aditi Kumar, Janae S. Stiffend, John S. Davis, Adegboyega K. Oyelere, Shafiq A. Khan

**Affiliations:** 1 Center for Cancer Research and Therapeutic Development, Clark Atlanta University, Atlanta, GA 30314, USA; scaggia@cau.edu (S.C.); smrruthivaidegi.venugopal@cshs.org (S.V.V.); 2318asg@gmail.com (A.S.G.); aditi.kumar.1216@gmail.com (A.K.); janae.stiffend@students.cau.edu (J.S.S.); 2 School of Chemistry and Biochemistry, Georgia Institute of Technology, Atlanta, GA 30318, USA; stapadar3@mail.gatech.edu (S.T.); bocheng.wu@gatech.edu (B.W.); 3 Department of Obstetrics and Gynecology, College of Medicine, University of Nebraska Medical Center and VA Medical Center, Omaha, NE 68198, USA; jsdavis@unmc.edu; 4 Parker H. Petit Institute for Bioengineering and Bioscience, Georgia Institute of Technology, Atlanta, GA 30318, USA

**Keywords:** Gα_i_2, cell migration, small molecule inhibitors, cancer, invasion

## Abstract

Heterotrimeric G-proteins are ubiquitously expressed in several cancers, and they transduce signals from activated G-protein coupled receptors. These proteins have numerous biological functions, and they are becoming interesting target molecules in cancer therapy. Previously, we have shown that heterotrimeric G-protein subunit alphai2 (Gα_i_2) has an essential role in the migration and invasion of prostate cancer cells. Using a structure-based approach, we have synthesized optimized small molecule inhibitors that are able to prevent specifically the activation of the Gα_i_2 subunit, keeping the protein in its inactive GDP-bound state. We observed that two of the compounds (**13** and **14**) at 10 μΜ significantly inhibited the migratory behavior of the PC3 and DU145 prostate cancer cell lines. Additionally, compound **14** at 10 μΜ blocked the activation of Gα_i_2 in oxytocin-stimulated prostate cancer PC3 cells, and inhibited the migratory capability of DU145 cells overexpressing the constitutively active form of Gα_i_2, under basal and EGF-stimulated conditions. We also observed that the knockdown or inhibition of Gα_i_2 negatively regulated migration of renal and ovarian cancer cell lines. Our results suggest that small molecule inhibitors of Gα_i_2 have potential as leads for discovering novel anti-metastatic agents for attenuating the capability of cancer cells to spread and invade to distant sites.

## 1. Introduction

Metastasis is the leading cause of mortality of patients with cancer. Metastatic transformation is a complex process, driven by a cascade of biological events—collectively defined as the metastatic cascade—which starts from the escape of the epithelial cells from the primary tumors, invasion throughout the surrounding extracellular matrix (ECM) and stromal cell layers, intravasation into the blood vessels, survival in the circulation, arrival to distant organ sites, extravasation into the parenchyma of the distant organs, the initial formation of micrometastasis, and starting of the proliferation at the metastatic sites, with a subsequent generation of neoplastic growths, known as “metastatic colonization” [1,2]. Several are the mechanism involved during metastatic progression and formation. One of the first process by which the cancer cells gain migratory and invasive properties is the epithelial-to-mesenchymal transition (EMT). Normal epithelial cells, from which cancer cells develop, are very tightly close to each other, through a very complex architecture of proteins and junctions, such as adherens junctions, desmosomes and tight junctions [3]. The EMT program involves downregulation of the proteins involved in cell-to-cell and cell-to-matrix adhesion, dissolution of adherens and tight junctions and a loss of cell polarity, to become motile [2]. Some of the players which have crucial roles during this metastatic transformation are the integrins, which have important functions during the intra- and extravasation processes [4].

As we previously stated, the initiation of metastasis begins with dissemination of tumor cells from the primary tumor to local and distant sites by a process called tumor cell motility [5]. Tumor cell motility or cell migration is induced by the activation of several receptors, including receptor tyrosine kinases (RTKs) and G-protein coupled receptors (GPCRs) [6,7,8,9]. The activation of GPCRs by chemokines and prostaglandins leads to the activation of a variety of heterotrimeric G proteins [10,11,12].

Several studies have implicated the role of Gα_i_ family members in cancer cell migration [13,14]. Previously, we have shown that Gα_i_2 plays a critical role in oxytocin (OXT) and EGF signaling, to induce the cell migration of prostate cancer cells [15]. We have also shown that Gα_i_2 acts at two distinct levels, to induce cell migration in prostate cancer cells. First, its activation through specific GPCRs is required to induce cell migration and invasion in response to several stimuli, such as chemokines, transforming growth factor β1 (TGFβ1) and OXT. These effects are, as expected, pertussis toxin (PT)-sensitive, and are upstream of the activation of PI3-kinase signaling pathway. Second, the activation of Gα_i_2 via GPCR is not required for cell migration induced by EGF, acting via its protein tyrosine kinase receptors. EGF induces migratory behavior in prostate cancer cells (PC3 and DU145), and these effects are not inhibited by pretreatment of the cells by PT [15,16]. However, knockdown and knockout of endogenous Gα_i_2 in prostate cancer cells induced an attenuation of EGF-dependent cell migration and invasion. Furthermore, these effects of Gα_i_2 are exerted down-stream of activation of PI3-kinase/AKT/mTOR/Rac1 pathway, and are required for formation of lamellipodia at the leading edge of migrating cells. This novel role of Gα_i_2 makes it an interesting therapeutic target to inhibit cell motility and metastasis [17,18,19].

The only commercially available inhibitor for the activation of Gα_i/0_ proteins is PT. PT catalyzes the ADP-ribosylation of the α subunits of the heterotrimeric G_i/o_ protein family, preventing the G proteins from interacting with their cognate G protein-coupled receptors [20]. However, as described above, it does not block the effects of Gα_i_2 on the lamellipodia formation and the induction of cell migration in response to stimuli, such as EGF, which do not act via GPCR. Therefore, there is a real urgency to develop small molecule inhibitors, which are useful for both pharmacological studies and the effective inhibition of monomeric subunits of Gα_i_2 protein.

Previously, it has been shown that small molecule inhibitors, targeting Gα_i_ and Gα_q_ subunits, act as guanine nucleotide dissociation inhibitors (GDI). These molecules were able to partially restore cyclic adenosine monophosphate (cAMP) levels in forskolin-stimulated cells [21]. These compounds are, however, weakly active, showing maximum inhibition of approximately 38% at 300 μM [21]. To demonstrate the feasibility of small molecule disruption of the function of Gα_i_2 protein as a strategy for mitigating cancer cell migration, we used one of the more synthetically tractable lead compounds, ketimine **9827** (referred to **12** in the following sections), as a template to develop more potent Gα_i_2 inhibitors. Subsequently, we screened compound **12** and the newly synthesized compounds for their effects on intracellular Gα_i_2 activity and migration of multiple cancer cell types. We observed that two of the new compounds, **13** and **14**, are more potent than **12** in inhibiting cell migration and preventing Gα_i_2 activation. Additionally, compound **14** was able (i) to block the activation of Gα_i_2 in oxytocin-stimulated prostate cancer PC3 cells; (ii) to induce the phosphorylation of CREB protein, downstream effector of cAMP production; (iii) to negatively regulate the migration of the renal and ovarian cancer cell lines. Together, these data confirmed the essential role of Gα_i_2 protein in mediating tumor cell migration, suggesting its potential as a molecular target for developing novel small molecule anti-metastasis agents, a new frontier in cancer therapy.

## 2. Results

### 2.1. Gα_i_2 Inhibitors Design: Molecular Docking Analysis and Synthesis

**Figure 1:**
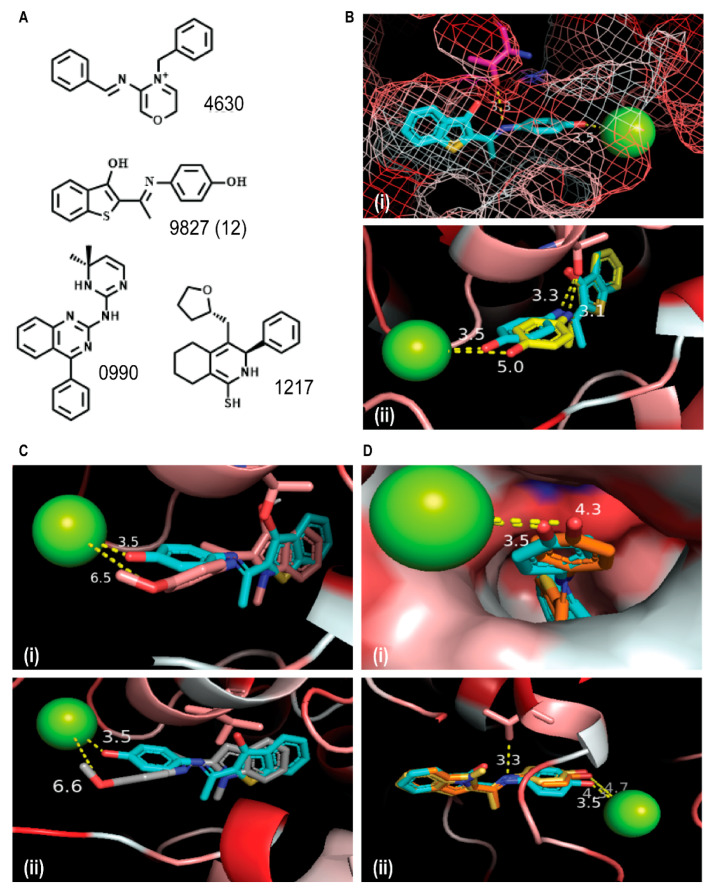
Structure-based design of Gα_i_2 Inhibitors. (**A**) Representative examples of Gα_i_1 Inhibitors. (**Bi**) Docked output of compound **9827** (**12**) at the active site of Gα_i_1–GDP, showing the placement of the thiophene-hydroxyl and benzothiophene sulfur groups. (**Bii**) Overlay of docked orientations of **12** in the presence (cyan) or absence (yellow) of Mg^2+^ ion. (**C**) Overlay of docked orientations of **12** (cyan) and **9a** (teal) (**Ci**), and **12** (cyan) and **9b** (grey) (**Cii**). (**Di**) Overlay of the docked outputs of **12** (cyan) and **13** (orange). (**Dii**) Overlay of the docked outputs of **12** (cyan), **13** (orange) and **14** (brown).

Of the four Gα_i_ selective inhibitors disclosed by Appleton et al. (Figure 1A) [21], ketamine **12** is the most synthetically tractable. To obtain structure-based information that may provide clues on how to further enhance the potency of **12**, we analyzed its docked poses at the active site of Gα_i_1–GDP (PDB: 2OM2) using Autodock Vina [22]. We observed that **12** adopted a low energy conformation, in which its thiophene-hydoxyl group is inserted in a hydrophobic pocket, although it may engage in H-bond interaction with a nearby hydroxyl group of Thr48 that is ideally oriented to the H-bond with its imine moiety (Figure 1Bi). However, this placement of the thiophene-hydoxyl group in the hydrophobic pocket may be counterproductive to the binding affinity of **12**, as it forces the benzothiophene sulfur group to be oriented in a pocket guarded by hydrophilic residues. So, we postulated that analogs of **12** having the thiophene-hydoxyl group deleted or replaced by small non-polar group could have enhanced binding affinity to Gα_i_. The other moiety of **12** that engages in productive interaction with the active site residues is its phenolic moiety, which interacts with the Mg^2+^ bound to GDP (Figure 1Bii). This may be the key interaction which stabilizes Gα_i_1–GDP, thereby preventing the exchange of GDP for GTP necessary for activation of Gα_i_. This observation suggests that modifications at the phenolic moiety of **12** may not be well tolerated.

To test our inferences from the docked poses of **12**, we designed compounds **9a, 9b**, **13** and **14**. Compounds **13** and **14** are analogs of **12** lacking the thiophene OH-group and with thiol- to N-methyl amino-group substitution, respectively. Compounds **9a** and **9b** are derivatives of **14**, designed to test the effect of modification to the phenolic group on Gα_i_2 inhibition activity. Analysis of the docked outputs of these compounds after molecular docking revealed interesting observations, which may corroborate several of our inferences. The methyl ether group in **9a** and **9b** essentially eliminates the possibility of their productive contact with the active site Mg^2+^, possibly depriving them of the interaction which stabilizes Gα_i_1–GDP (Figure 1C). Compound **13** and **14** adopt low energy docked orientations with their phenolic groups, occupying positions that are nearly identical to that occupied by the phenolic group of **12** (Figure 1Di). The deletion of the thiophene-hydoxyl group in **13** forces its benzothiophene to adopt an orientation, where its sulfur group is now placed in the hydrophobic pocket occupied by the thiophene-hydoxyl group of **12**. Additionally, the N-methyl amino-group of the benzopyrrole moiety of **14** is similarly oriented as the benzothiophene sulfur **13**, and presumably fits better into the hydrophobic pocket (Figure 1Dii, yellow for **14**, orange for **13**). The overlay of the docked outputs of **12**, **13** and **14** revealed that the benzothiophene ring of **13** and the benzopyrrole ring of **14** adopt orientations where their sulfur and N-methyl amino groups, respectively, are placed in the hydrophobic pocket occupied by the thiophene-hydoxyl group of **12**. Based on these docking results, we expect **13** and **14** to have similar or enhanced Gα_i_ inhibition activities compared to **12**, while **9a** and **9b** are expected to be considerably weaker.

**Figure 2:**
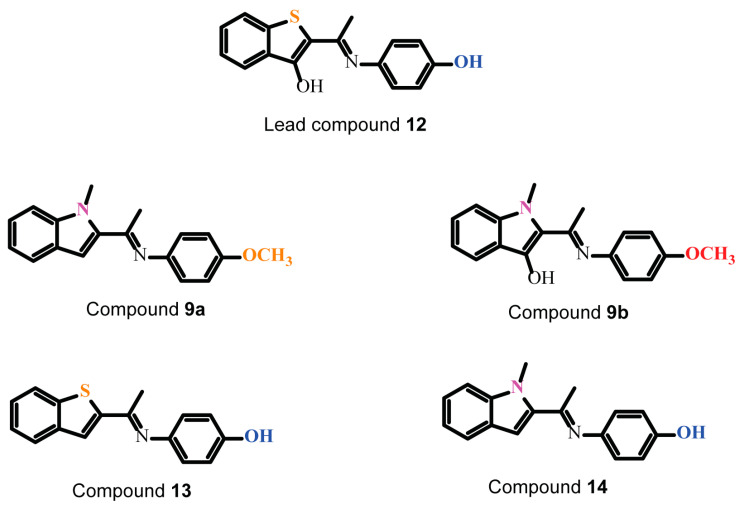
Chemical structure of the Gα_i_2 Inhibitors. Compounds **13** and **14** are analogs of ketamine **12** lacking the thiophene OH-group and with thiol- to N-methyl amino-group substitution, respectively. Compounds **9a** and **9b** are derivatives of **14,** designed to test the effect of modification to the phenolic group on Gα_i_2 inhibition activity.

To verify these in silico predictions, we synthesized lead compound **12** and compounds **9a**, **9b**, **13** and **14**, following the reaction routes in Figure S1. Briefly, ketimines **9a, 9b, 10**–**11** were synthesized from the corresponding methylketones **1**–**4** and anisidine (**6**) or *O*-silyl-protected *p*-hydroxyaniline (**5** and **7**) using catalytic amount of *p*-TsOH and toluene as solvent [23]. The reactions were performed in Dean-Stark apparatus to remove water and resulting in the target compounds in low to moderate yields. Subsequently, CsF-mediated deprotection of the silyl protection groups of intermediates **8**, **10** and **11** furnished the requisite compounds **12**, **13** and **14** (details of the general procedures to prepare ketimines are in the Supplementary Materials section). A panel of the basic chemical structure of the lead compound and the newly synthesized small molecules is presented in Figure 2.

Compounds were then screened in assays to determine their effects on the intracellular Gα_i_2 activity and migration of selected cancer cell lines.

### 2.2. Inhibition of Gα_i_2 Activation Decreases the Migration and Invasion in Prostate Cancer Cells

Previously, we found that endogenous Gα_i_2 is essential for cell migration and invasion in prostate cancer cells in response to different stimuli, such as EGF, OXT, TGFβ1 and SDF-1α [15,17]. To determine the physiological effects of the newly synthesized small molecules, we performed transwell migration assays in PC3 cells, using the inhibitors at three different concentrations (10, 50 and 100 μM). The lead compound **12** only caused a reduction of the migratory capability of PC3 cells in the presence and absence of EGF stimulus at 50 and 100 μM, but no effect at 10 μM (Figure S2A). We also observed that compounds **9a** and **9b** slightly decreased the migratory capability of PC3 cells at 100 μM, but not at 50 or 10 μM (Figure S2B,C). Whereas, at the concentrations of 10, 50 and 100 μM, compounds **13** and **14** were able to reduce the migratory capability of PC3 cells in presence of EGF, compared with the control cells (Figure S2D,E). In addition, we also performed cell viability assays for all the tested compounds at several concentrations and compounds **12**, **13** and **14** were cytotoxic at 50 μM and 100 μM, but had no effect on cell viability at 10 μM. Compounds **9a** and **9b** didn’t affect cell viability even at 100 μM.

Based on these results we used compounds **13** and **14** at 10 μM concentrations in all further experiments, and we used compound **9b** as a negative control.

**Figure 3:**
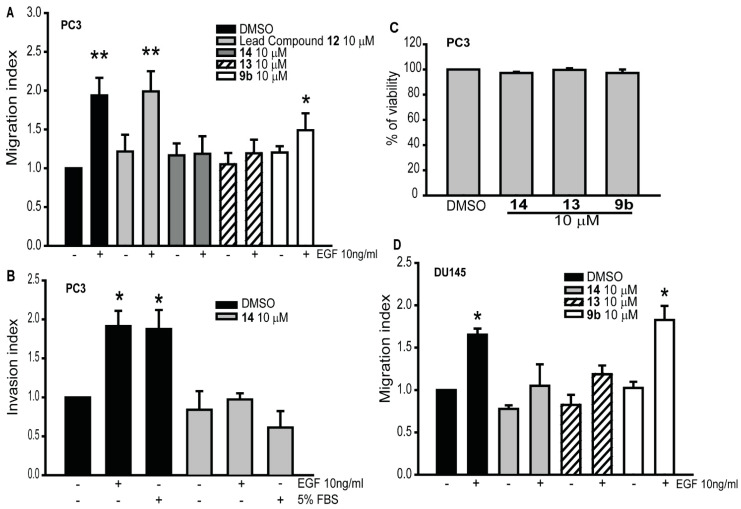
Migratory and invasive capability of prostate cancer cells is differently modulated by the new Gα_i_2 small molecules inhibitors. (**A**) PC3 cells were incubated with or without the lead compound and three different inhibitors (**9b**, **13** and **14**) at the final concentration of 10 μM, and then subjected to transwell migration assays in the presence (+) or absence (−) of the epidermal growth factor (EGF) (10 ng/mL). The results are expressed as migration index. Each bar represents mean ± SEM. (**B**) The invasive behavior of PC3 cells treated with or without compound 14 at the final concentration of 10 μM in the presence (+) or absence (−) of EGF (10 ng/mL). The results are expressed as invasion index. Each bar represents mean ± SEM. 5% FBS was used as positive control. (**C**) PC3 cells were treated with the inhibitors, at a final concentration of 10 μM. MTS assays were conducted, and the results were expressed as % of viable treated cells against the control cells. Each bar represents mean ± SEM. (**D**) DU145 cells were incubated with or without the three inhibitors (**9b**, **13** and **14**) at the final concentration of 10 μM, and then subjected to transwell migration assay in the presence (+) or absence (−) of EGF (10 ng/mL). Results are expressed as migration index. Each bar represents mean ± SEM (* *p* ≤ 0.05; ** *p* ≤ 0.01).

At 10 μM, compounds **9b** and **12** had no effect on migration of PC3 cells in the presence of EGF (average cell number per insert ± Standard Error: 265.5 ± 9.66 and 357.25 ± 31.95, respectively. Control cells: 186.5 ± 21.68; EGF treated cells: 352.75 ± 39.4). However, compounds **13** and **14** significantly decreased EGF-induced migratory capability (211.25 ± 4.3 and 206.75 ± 23.73, respectively) (Figure 3A), an observation that is in agreement with our in silico docking prediction. To determine if these small molecules are also able to inhibit the invasive capability of PC3 cells, we performed invasion assays, using one of the most effective compound, **14**. As shown in Figure 3B, the invasive capability of PC3 cells was significantly reduced in the presence of the compound in response to both EGF and FBS (586.75 ± 34.38 and 568.33 ± 61, respectively. Control cells: 654.5 ± 91.41; EGF treated cells: 1122.5 ± 153.8; 5%FBS treated cells: 1345.25 ± 91). Compounds **9b**, **13** and **14** did not affect cell viability at the concentration of 10 μM (Figure 3C). We also tested the effects of these three inhibitors **9b**, **13** and **14** on cell migration in DU145, an additional prostate cancer cell line. As shown in Figure 3D, compounds **13** and **14** caused inhibition of the migratory capability of EGF-induced DU145 cells (108 ± 24.17 and 91.8 ± 11.48, respectively. Control cells: 96.2 ± 18.37; EGF treated cells: 199.4 ± 31). On the other hand, compound **9b** had no effect on EGF-induced cell migration (185 ± 39).

### 2.3. Compound 14 Blocks Activation of Gα_i_2

**Figure 4:**
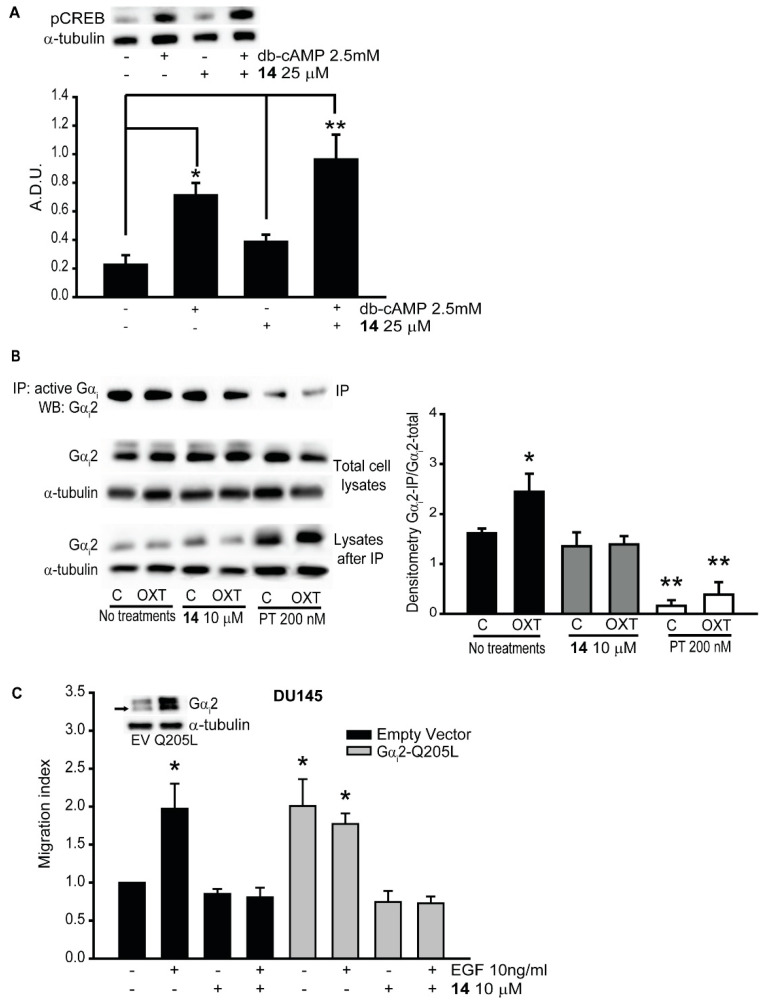
The inhibitors blocked the activation of Gα_i_2. (**A**) PC3 cells were pre-treated with (+) or without (−) compound **14** at 25 μM and then stimulated with (+) or without (−) dibutyryl-cAMP (dbcAMP) at 2.5 mM. Total cell lysates were subjected to Western blot analysis, using the pCREB (Ser129) antibody. Independent experiments were conducted at least three times, and representative images of immunoblots are shown. Densitometr analysis was performed using ImageJ [24]. (**B**) Total cell lysates from different treatments were immunoprecipitated using anti-active Gα_i_ antibody, and the immunoprecipitates were immunoblotted with anti-Gα_i_2 antibody. Independent experiments were conducted three times, and representative images of immunoblots are shown. Densitometric analysis was performed using ImageJ [24]. (**C**) Cell migrations in parental DU145-EV and DU145-Gα_i_2-Q205L cells were performed after incubation with (+) or without (−) compound **14** at 10 μM, in presence (+) or absence (−) of EGF (10 ng/mL). Results are expressed as migration index. Each bar represents mean ± SEM (* *p* ≤ 0.05; ** *p* ≤ 0.01).

Endogenous Gα_i_ proteins inhibit cAMP synthesis and signaling, therefore we incubated PC3 cells with compound **14** (25 μM) for one hour, and then stimulated with dibutyryl-cAMP (dbcAMP), a cell-permeable cAMP analog, at 2.5 mM for ten min. Western blot analysis for phosphorylated cyclic AMP response element-binding protein (pCREB) was performed. We observed an increase in the amount of pCREB in PC3 cells treated with Gα_i_2 inhibitor, compared to the control (Figure 4A), suggesting reduced Gα_i_ activity in these cells. Then, we incubated PC3 cells with compound **14** (10 μM) for 30 min and then we treated the cells with EGF (10 ng/mL) or OXT (200 nM) for additional 30 min. We performed immunoprecipitation using anti-active Gα_i_ antibody, and we conducted Western blot analysis using a specific anti-Gα_i_2 antibody. We observed that, after treatments with OXT, the levels of active Gα_i_2 were increased, compared to the controls. Moreover, in the presence of compound **14**, the levels of active Gα_i_2 were significantly reduced after stimulation with OXT, compared to the controls. We used PT treatments as positive controls, which caused a significant reduction in the levels of active Gα_i_2 in both control and OXT-stimulated cells, as shown in the quantitative analysis graph (Figure 4B, right panel).

Next, we overexpressed constitutively active form of Gα_i_2 (Gα_i_2-Q205L) in DU145 cells and determined the effects of the inhibitors on cell migration in these cells. As shown in Figure 4C, overexpression of Gα_i_2-Q205L in DU145 cells led to significant increase in cell migration, which was not further increased in the presence of EGF (Control Q205L cells: 153 ± 14; EGF treated Q205L cells: 160.4 ± 5.24), compared to the cells transfected with empty vectors (DU145-EV) (control EV cells: 93.4 ± 9.46; EGF treated EV cells: 260 ± 9.46). Treatments with inhibitor **14** (10 μM) resulted in the attenuation of basal and EGF-stimulated cell migration in DU145 cells overexpressing constitutively active Gα_i_2 (Gα_i_2-Q205L) (73 ± 7.6 and 93.2 ± 14.17, respectively) (Figure 4C).

### 2.4. Gα_i_2 Protein is Essential for Cell Migration in Renal and Ovarian Cancer Cells

**Figure 5:**
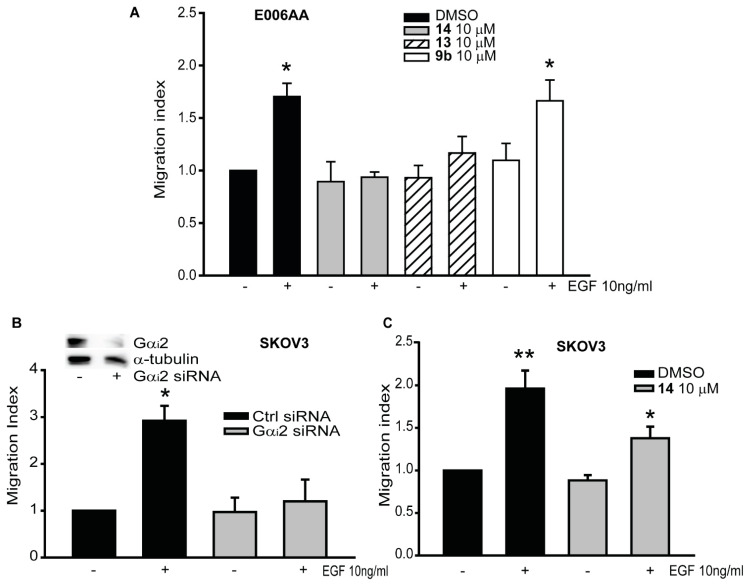
Inhibition of Gα_i_2 decreased migratory capability of renal, breast and ovarian cancer cells. (**A**) E006AA cells were subjected to transwell migration assay, incubated with or without the three inhibitors (**9b**, **13** and **14**) at the final concentration of 10 μM, in the presence (+) or absence (−) of EGF (10 ng/mL). The results are expressed as migration index. Each bar represents mean ± SEM. (**B**) SKOV3 cells were transfected with control and Gα_i_2 siRNAs and then subjected to transwell migration assays in the presence (+) or absence (−) of EGF (10 ng/mL). Results are expressed as migration index. Each bar represents mean ± SEM. (**C**) SKOV3 cells were incubated with or without compound **14** at 10 μM, and then subjected to transwell migration assay in the presence (+) or absence (−) of EGF (10 ng/mL). The results are expressed as migration index. Each bar represents mean ± SEM (* *p* ≤ 0.05; ** *p* ≤ 0.01).

Previously, we have shown the essential role of the Gα_i_2 protein in the migration of prostate cancer cell lines, including E006AA cells, which have recently been found to be renal cancer cells [15,17]. In E006AA cells, compounds **13** and **14** caused the inhibition of the migratory capability of EGF-induced cell migration at 10 μM (952.66 ± 62.75 and 844 ± 81.36, respectively. Control cells: 810 ± 115.6; EGF treated cells: 1443 ± 175.21). On the other hand, compound **9b** at the same concentration had no effects on EGF-induced cell migration (1324.6 ± 168.2) (Figure 5A). To determine whether Gα_i_2 plays a similar role in other cancers, we performed migration assays using SKOV3, ovarian cancer cell lines. In SKOV3, the knock-down of Gα_i_2 protein resulted in significant reduction in the number of migrating cells in EGF treated cells, (SKOV3 treated with EGF: 490.25 ± 64.79) compared with the cells transfected with control siRNA (SKOV3 control cells: 177.5 ± 37.23) (Figure 5B). As expected, treatments with compound **14** (10 μM), also impaired the migratory capability of SKOV3 cell lines, in EGF stimulated cells (control cells: 202.5 ± 29.43; SKOV3 cells treated with EGF: 470 ± 54.12) (Figure 5C).

## 3. Discussion

Metastatic dissemination is one of the main causes of recurrence and death from cancer, and it is regulated by the activation of several mechanisms and pathways [6,25,26]. Treatments that inhibit the cell motility or proteins involved in the enhancement of cell migration represent an interesting and attractive approach for controlling metastatic dissemination. However, very few drugs able to inhibit cell migration, have been tested in clinical trials [27].

Recently, Appleton et al. identified small molecule GDIs which weakly inhibit Gα subunits at high micromolar concentrations while maintaining intact the stimulation of the Gβγ signaling [21]. In the present study, we have designed, synthesized and profiled the effects of four compounds (**9a**, **9b**, **13** and **14**) on the migration of selected cancer cell lines. These compounds were designed based on compound **12** (ketamine **9827**), the more synthetically tractable compound, as disclosed by Appleton et al. [21]. At this time, there is no suitable crystal structure of Gα_i_2 that can be used for our purpose. Furthermore, according to Clustal 0 (1.2.4) (https://www.ebi.ac.uk/Tools/msa/clustalo/) multiple sequence alignment, Gα_i_1 and Gα_i_2 proteins have more than 90% amino acid sequence similarities and their GTP-binding sites are conserved (Figure S3). For all these reasons, to understand the structural basis of the interaction of compound **12** and the newly synthesized compounds with Gα_i_, we performed molecular docking studies, using the crystal structure of Gα_i_1-GDP (PDB: 2OM2), using Autodock Vina.

To determine the efficacy of the newly synthesized compounds, we screened the lead compound (ketamine **12**) and compounds **9a**, **9b**, **13** and **14**, using several cancer cell types. These compounds impair the activation of Gα_i_2 by inhibiting the conversion of GDP- to GTP-state of the Gα_i_2 subunit. A similar inhibition of GDP-GTP exchange by small molecules targeting Rho GTPases has been shown by others to impair cancer progression and invasion [28,29]. Among all the compounds that we investigated, **13** and **14**, at the concentration of 10 µM, were able to significantly reduce the migratory capability of prostate cancer cells (Figure 3). The enhanced inhibition of cell migration displayed by compound **13** and **14** confirms their efficacy, matching the results from the docking studies, which showed that these two molecules are the most active (Figure 1). These results suggest that these new small molecule inhibitors are able to significantly reduce migration and invasion in prostate cancer models, and concur with our previous findings, where the knockdown of endogenous Gα_i_2 protein significantly decreased the migratory capability of prostate cancer cell lines [15,17].

Gα_i_ proteins inhibit the stimulation of the adenylate cyclase, leading to a decrease of cAMP. The function of this second messenger is to activate PKA, which leads to an increase in the phosphorylation of cAMP response element binding protein (CREB). When Gα_i_ is active, the levels of pCREB are decreased [30]. Indeed, after treatments of PC3 cells with compound **14**, the levels of pCREB were increased after stimulation with dibutyryl-cAMP, a permeable analog of cAMP, compared to the control cells (Figure 4A). We also performed immunoprecipitation with active Gα_i_ antibody, to evaluate the effects of compound **14**. The results showed that the levels of active Gα_i_2 were reduced after stimulation with OXT in the presence of compound **14**, compared to the controls (Figure 4B). We used OXT, because this hormone stimulates the activation of Gα_i_ proteins and also, we previously found that OXT induces migration of prostate cancer cells by activating Gα_i_2 [17]. Finally, when we overexpressed constitutively active form of Gα_i_2 (Gαi2-Q205L) in DU145 cells, which significantly increased cell migration without exogenous stimulation, we observed that compound **14** significantly reduced migration in DU145-Gαi2-Q205L cells (Figure 4C). Compound **14** inhibits the activation of Gα_i_2 by competing with the GTP at its binding site at 10 μΜ. These results match other studies in which the small molecule inhibitor NSC23677, targeting the small GTPase Rac1, was able to reduce the migratory capability of PC3 prostate cancer cells overexpressing constitutively active form of Rac1 [31,32].

The epidermal growth factor (EGF) is one of the most common inducer of migration of normal and cancer cells [28,32,33]. We also know that cell migration elicited by tyrosine kinase receptors is not mediated by G-proteins. In our previous studies, however, we demonstrated that the absence of the Gα_i_2 protein impaired the migratory capability of several cancer types, when EGF was used as chemotactic inducer, revealing a novel mechanism that need to be evaluated. Additionally, we showed that the impaired migration caused by the absence of Gα_i_2 was independent of Rac1 activation. We concluded that Gα_i_2 protein was acting independent or downstream of PI3K/AKT/Rac1 signaling pathway [17]. For all these reasons, we used EGF to induce migration in the cellular models we investigated in this study.

The role of Gα_i_2 protein is essential in the cell migration and invasion of prostate cancer cells [15,17]. To determine if the Gα_i_2 protein is also required for migration in other cancer cell types, we silenced the Gα_i_2 expression in SKOV3 ovarian cancer cells, and we observed that, indeed, the migratory capability of these cells was also significantly reduced. Importantly, we also observed that compound **14** significantly reduced migration of aforementioned cell lines. These results lead us to conclude that Gα_i_2 is an important player in the regulation of cancer cell migration, and that is something that can be investigated to understand the mechanisms that regulate the process.

## 4. Materials and Methods

### 4.1. Chemicals and Reagents

Anhydrous solvents and other reagents were purchased from Sigma-Aldrich (St. Louis, MO, USA) and VWR International, (Radnor, PA, USA), and were used without further purification. Analtech silica gel plates (60 F254) were utilized for analytical thin-layer chromatography (TLC), and Analtech preparative TLC plates (UV254, 2000 μm) were used for purification. Silica gel (200–400 mesh) was used in column chromatography. TLC plates were visualized using UV light, anisaldehyde, and/or iodine stains. NMR spectra were obtained on a Varian-Gemini 400 MHz and Bruker Ascend™ 500 and 700 MHz magnetic resonance spectrometer. ^1^H NMR spectra were recorded in parts per million (ppm) relative to the residual peaks of CHCl_3_ (7.24 ppm) in CDCl_3_ or CHD_2_OD (4.78 ppm) in CD_3_OD or DMSO-*d*_5_ (2.49 ppm) in DMSO-*d*_6_. MestReNova (version 11.0) was used to process the original “fid” files. High-resolution mass spectra were gathered with the assistance of the Georgia Institute of Technology mass spectrometry facility (Atlanta, GA, USA).

Anti-α-tubulin and bovine serum albumin (BSA) were obtained from Sigma-Aldrich (St. Louis, MO, USA). Rat tail collagen, Matrigel and transwell inserts were obtained from BD Biosciences (San Jose, CA, USA). DAPI (4′,6-Diamidino-2-Phenylindole, Dilactate) was purchased from Invitrogen through Thermo Fisher Scientific (Eugene, OR, USA). Rabbit polyclonal anti-Gα_i_2 antibody (sc-7276), control and Gα_i_2 siRNAs, and transfection reagents (sc-295228) were purchased from Santa Cruz Biotechnology (Dallas, TX, USA). Rabbit polyclonal anti-CREB (phospho-Ser129) antibody (#11273) was purchased from Signalway Antibody LLC (College Park, MD, USA). The epidermal growth factor (EGF) was obtained from Life Technologies (Grand Island, NY, USA). The anti-active Gα_i_ antibody was purchased from NewEast Biosciences (Malvern, PA, USA). The anti-rabbit and anti-mouse immunoglobulins coupled with horseradish peroxidase (IgG-HRP), were obtained from Promega (Madison, WI, USA). Cell culture reagents were obtained from Corning Life Sciences (Tewksbury, VA, USA). pcDNA3.1 control vector and vector encoding the constitutively active form of Gα_i_2 (pcDNA3.1-EV and pcDNA3.1-Gα_i_2-Q205L, respectively) were purchased from cDNA Resource Center (Bloomsburg, PA, USA).

### 4.2. Cell Culture

Human prostate cancer cell lines (DU145 and PC3) were obtained from American Type Culture Collection (ATCC) (Rockville, MD, USA). DU145 and PC3 are androgen independent cell lines, derived from brain and bone metastatic sites, respectively. They were maintained in Minimum Essential Medium, supplemented with 5% FBS, in a 5% CO_2_ environment at 37 °C, as previously described [15,17,34,35]. E006AA cells were kindly provided by Dr. Shahriar Koochekpour (Roswell Park Cancer Institute, Buffalo, NY, USA). These cells were maintained and cultured as described previously [36,37]. Human ovarian adenocarcinoma cell line SKOV3 was obtained from American Type Culture Collection (ATCC) (Rockville, MD, USA) and maintained in Dulbecco’s Modified Eagle Medium, supplemented with 5% FBS, in a 5% CO_2_ environment at 37 °C.

### 4.3. Small Molecules Preparation and Docking

Molecular docking was performed on crystal structure of Gα_i_1–GDP bound to the Goloco Motif of Rgs14 (PDB: 2OM2) [38] using Autodock Vina [22], run through PyRx to manage the workflow and PyMol to visualize the results, as described previously [39,40]. Prior to docking, the water molecules and RGS14 protein motif were removed. Ligands were prepared by generating an energy minimized 3D structure in ChemBioDraw3D (Ultra 13.0). This was followed by processing with Autodock Tools 1.5.4. Docking runs were performed within a 25–30 Å cubic search space surrounding the binding pocket in the presence and absence of active site Mg^2+^ ion through PyRx. To ensure the results are comparable, the docking results we chose are the models with the highest binding affinity and similar orientation as lead compound 12.

Details about compound synthesis and characterization are provided in the supplementary information. For biological assays, the compounds were dissolved in DMSO at a starting concentration of 0.05 M (compound **12**) and 0.1 M (compounds **9a, 9b**, **13** and **14**), and then diluted in culture media to the final concentrations used for the assays. Dilutions of DMSO were used as controls.

### 4.4. Immunoprecipitation of Active Gα_i_

PC3 cells (3 × 10^6^ cell/dish) were incubated with or without inhibitor **14** (10 μM) for 30 min and then treated with EGF (10 ng/mL) and OXT (200 nM) for additional 30 min. Cells were lysed in ice-cold cell lysis buffer (Cell Signaling Technology, Danvers, MA, USA), and snap-frozen in liquid nitrogen. Total cell lysates, containing approximately 1000 μg of proteins, were used for immunoprecipitation using procedures described previously [41]. Briefly, lysates were incubated with 1 μg of anti-active Gα_i_ antibody, overnight at 4 °C. Immunocomplexes were collected by centrifugation after incubation with protein A/G-Sepharose beads for 48 h (Santa Cruz Biotechnology, Dallas, TX, USA), and were analyzed by Western blot analysis with specific anti-Gα_i_2 antibody (1:5000 dilution, ab157204, Abcam, Cambridge, MA, USA).

### 4.5. Transient Transfection with Constitutively Active Gα_i_2-Q205L Plasmid

DU145 cells were seeded in 6-well plates at a density of 2.0 × 10^5^ cells per well and transfected with pcDNA3.1-EV and pcDNA3.1-Gα_i_2-Q205L, using ViaFect™ transfection reagent (Promega, Madison, WI, USA), according to the manufacturer’s protocol. Briefly, media with no antibiotics (200 µL/well) containing 2 µg of plasmids DNA were mixed with the transfection reagent (6 µL/well) and, after 20 min, the mixtures were added drop by drop on the cells, and the cells were cultured for 48 h. Then, the cells were harvested and used for different assays.

### 4.6. Western Blot Analysis

Western blot analyses were performed as described previously [15,17,34,36]. Briefly, protein samples (30–35 µg proteins) were separated on 10% SDS-PAGE gels and transferred to polyvinylidene difluoride (PVDF) membranes (Millipore Corp., Bedford, MA, USA). After blocking, the membranes were incubated with different primary antibodies, at appropriate dilutions (1:1000 for pCREB; 1:500 for Giα2; 1:3000 for α-tubulin) overnight at 4 °C. After washing, the blots were incubated with appropriate secondary antibodies and developed in ECL mixture, using Syngene PXi Imaging System, according to the manufacturer’s manual. α-tubulin was used as loading control (Figures S4–S7).

### 4.7. Cell Viability Assay

Cell viability assays were performed using CellTiter 96^®^ AQueous One Solution Cell Proliferation Assay (MTS) from Promega (Madison, WI, USA), according to the manufacturer’s protocol. Briefly, 5.0 × 10^4^ cells/well were plated in a 96 well plates and incubated in a 5% CO_2_ environment at 37 °C overnight. After 24 h, the medium was replaced with fresh medium, containing different compounds at the appropriate concentrations. Diluted DMSO was used as a control. MTS assays were performed after 24 h and the absorbance read at 490 nm, using a spectrophotometer.

### 4.8. Cell Migration and Invasion Assays

In vitro cell migration and invasion assays were conducted using 24-well transwell inserts (8 μm), as described previously [15,17,34,36]. Briefly, transwell inserts were coated with rat tail collagen (50 mg/mL), for migration assays, and with 50 μL of a 1:4 Matrigel/coating buffer solution, for invasion assays. Cells were suspended at the appropriate density in appropriate media, and treated with the different inhibitors, at specific concentrations. For migration assays, EGF was used as chemoattractant (10 ng/mL) for PC3, DU145, E006AA and SKOV3 cells. The plates were incubated at 37 °C for 5 h (DU145, PC3 and SKOV3), and 24 h (E006AA) for migration assays, and 48 h for invasion assays. The nonmigratory cells were then removed using a cotton swab, and the outer cells were fixed and stained with 3 ng/mL of DAPI. Images of five non-overlapping fields of each insert were captured using Axiovert 200 M, Carl Zeiss (Thornwood, NY, USA) microscope, with a 10× magnification objective, and the number of stained nuclei were determined with automatic counting, using image analysis software (ZEN 2012; Carl Zeiss, https://www.zeiss.com/microscopy/int/downloads.html). Results were expressed as migration or invasion index defined as the average number of cells per field for test substance/the average number of cells per field for the medium control.

### 4.9. Statistical Analysis

All experiments were repeated at least three times using different cell preparations. The results are presented as mean ± SEM of at least three independent experiments and images from a single representative experiment are presented. One-way and two-way ANOVA analyses were employed, to assess the significance of differences among various treatment groups (*p* < 0.05), represented by asterisks: one asterisk represents that the data are significant against the controls, double asterisks represent the data are significant against the control and various treatment groups.

## 5. Conclusions

In conclusion, we disclosed new small molecules which target Gα_i_2, resulting in the inhibition of the migratory behavior of several cancer types. Of the compounds synthesized, **13** and **14** are the most effective at reducing motility of prostate, renal, and ovarian cancer cell lines. These compounds may be considered as potential leads for the development of new therapeutic modality for highly metastatic cancers.

Based on several studies, there are not consistent results regarding the expression of Gα_i_2 in primary and metastatic tumors. However, our findings indicate that targeting Gα_i_2 may be an effective therapy for many metastatic cancers, findings that will be further confirmed using these molecules in in vivo Xenograft metastatic models.

## Supplementary Materials

The following are available online at https://www.mdpi.com/2072-6694/12/6/1631/s1, Figure S1: Synthesis of ketimines 9a, 9b, 12, 13 and 14; Figure S2: Optimization of the dosage of the small molecule inhibitors, using PC3 cell lines; Figure S3: Alignment of Gα_i_1 and Gα_i_2 proteins, using Clustal Omega; Figure S4: Full Western blot images, related to the representative data of the Immunoprecipitation experiments; Figure S5: Full Western blot images, related to the representative data of the levels of p-CREB (A) and α-tubulin (B), after treatments with compound 14, in presence of db-cAMP; Figure S6: Full Western blot images, related to the representative data of the overexpression and knockdown of Gα_i_2 protein experiments in DU145 (Gα_i_2-Q205L) and SKOV3; Figure S7: Densitometric analysis of Western blot images.
