# Supplementary material for: Small Molecule Inhibitors Targeting Gαi2 Protein Attenuate Migration of Cancer Cells
Source: Cancers (Basel). 2020 Jun 19;12(6):1631. doi: 10.3390/cancers12061631 (PMC7353059; doi:10.3390/cancers12061631)
Supplement: Supplementary file 1 [file cancers-12-01631-s001.pdf]

Supplementary Materials

Small molecule inhibitors targeting Gα_i2 protein attenuate migration of cancer cells

Silvia Caggia[1], Subhasish Tapadar[2], Bocheng Wu[2], Smrruthi V. Venugopal[1^], Autumn S. Garrett[1], Aditi Kumar[1], Janae S. Stiffend[1], John S. Davis[4], Adegboyega K. Oyelere[2,3*], Shafiq A. Khan[1*]

[1]Center for Cancer Research and Therapeutic Development, Clark Atlanta University, Atlanta, 30314, GA, USA; [2]School of Chemistry and Biochemistry and [3]Parker H. Petit Institute for Bioengineering and Bioscience, Georgia Institute of Technology, Atlanta, 30318, GA, USA; [4]Department of Obstetrics and Gynecology, College of Medicine, University of Nebraska Medical Center, and VA Medical Center, Omaha, 68198, NE, USA; ^ Present address: Department of Surgery, Cedar-Sinai Medical Center, Los Angeles, 90048, CA, USA

***Correspondence:**

Shafiq A. Khan, Ph.D.
Center for Cancer Research and Therapeutic Development
Clark Atlanta University
223 James P. Brawley Dr, SW
Atlanta, GA 30314
Tel: 404-880-6795
E-mail: skhan@cau.edu

Adegboyega K. Oyelere, Ph.D.
School of Chemistry and Biochemistry and Parker H. Petit Institute for Bioengineering and Bioscience,
Georgia Institute of Technology
901 Atlantic Drive
Atlanta, GA 30332-0400
E-mail address: aoyelere@gatech.edu

Supplementary materials

Chemistry

Materials. Anhydrous solvents and other reagents were purchased either from Sigma or VWR and were used without further purification. Analtech silica gel plates (60 F254) were utilized for analytical TLC, and Analtech preparative TLC plates (UV254, 2000 μm) were used for purification. Silica gel (200−400 mesh) was used in column chromatography. TLC plates were visualized using UV light, anisaldehyde, and/or iodine stains. NMR spectra were obtained on a Varian-Gemini 400 MHz and Bruker Ascend™ 500 and 700 MHz magnetic resonance spectrometer. ^{1}H NMR spectra were recorded in parts per million (ppm) relative to the residual peaks of $CHCl_3$ (7.24 ppm) in $CDCl_3$ or CHD_2OD (4.78 ppm) in CD_3OD or DMSO-d_5 (2.49 ppm) in DMSO-d_6. MestReNova (version 11.0) was used to process the original "fid" files. High-resolution mass spectra were gathered with the assistance of the Georgia Institute of Technology mass spectrometry facility (Atlanta, GA). *N*-methyl-2-acetylindole (**1**)[1,2], 1-(3-hydroxy-1-methylindol-2-yl)ethanone (**2**)[3], 4-((*tert*-butyldiphenylsilyl)oxy)aniline (**5**)[4], and 4-((*tert*-butyldimethylsilyl)oxy)aniline (**7**)[5] were synthesized adapting literature protocols.

General Procedure for Preparation of *Ketimines* (*Method A*). A solution of the corresponding methylketones **1-4** (1 mmol), corresponding amines **5-7** (1.2 mmol) and *p*-toluenesulfonic acid monohydrate (5 mol%) in anhydrous toluene (5 mL) was heated under reflux with a Dean-Stark trap for 10 hour, then cooled and neutralized by addinh saturated aqueous $NaHCO_3$ solution and then organic layer was separated. The aqueous layer was further extracted with ethyl acetate (20 mL) and then the combined organic layers was washed with water (10 mL), brine (10 mL), dried over anhydrous Na_2SO_4, filtered and evaporated to dryness. The residue was purified by preparative chromatography with a hexane-ethyl acetate mixture as mobile phase to give the ketimine compounds **8**-**11**.

General Procedure for silyl deprotection (*Method B*). Silyl protected ketimine compounds **8**, **10**, and **11** were dissolved in 2:1 MeOH-THF and then CsF (2 equiv.) was added to the solution and the resultant solution was stirred for1 h. The reaction was quenched by adding water and was extracted with ethyl acetate (20 mL) and the

aqueous layer was separated. Ethyl acetate layer was washed with brine (10 mL), dried over anhydrous Na_2SO_4, filtered, and concentrated to dryness. The residue was purified by preparative chromatography with a hexane-ethyl acetate mixture as mobile phase to give the target molecules **12-14**.

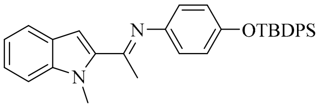

(*E*)-*N*-(4-((*tert*-butyldiphenylsilyl)oxy)phenyl)-1-(1-methyl1*H*-indol-2-yl)ethan-1-imine **8**. Method A; purified by preparative chromatography using 5% ethyl acetate-hexane mixture as mobile phase. Yellow oil; yield: 15%. ^{1}H NMR (400 MHz, $CDCl_3$) *δ* 7.75 – 7.66 (m, 4H), 7.64 – 7.55 (m, 1H), 7.45 – 7.31 (m, 6H), 7.28 (t, *J* = 7.6 Hz, 1H), 7.23 – 7.18 (m, 1H), 7.13 – 7.05 (m, 1H), 6.97 (s, 1H), 6.78 – 6.69 (m, 2H), 6.57 (d, *J* = 8.3 Hz, 2H), 4.14 – 4.00 (s, 3H), 2.23 (s, 3H), 1.09 (s, 9H).

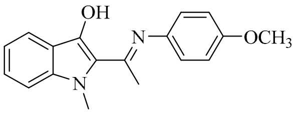

(*E*)-2-(1-((4-methoxyphenyl)imino)ethyl)-1-methyl-1*H*-indol-3-ol **9a**. Method A; purified by preparative chromatography using 40% ethyl acetate-hexane mixture as mobile phase. Orange solid; yield: 15%. ^{1}H NMR (700 MHz, $CDCl_3$-*d*) δ 7.81 (d, *J* = 7.7 Hz, 1H), 7.40 (t, *J* = 7.6 Hz, 1H), 7.09 (dd, *J* = 13.1, 8.2 Hz, 3H), 6.94 (t, *J* = 7.3 Hz, 1H), 6.89 (d, *J* = 8.4 Hz, 2H), 3.80 (s, 3H), 3.49 (s, 3H), 2.40 (s, 3H). ^{13}C NMR (176 MHz, $CDCl_3$) *δ* 177.58, 157.76, 150.15, 149.89, 131.60, 131.45, 126.43, 123.41, 123.17, 121.53, 118.80, 114.70, 114.66, 110.93, 55.72, 35.93, 17.07. HRMS (EI) m/z Calcd. for $C_{18}H_{18}O_2N_2$ $[M]^+$: 294.1371, found 294.1368.

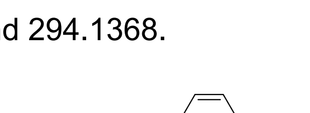

(*E*)-*N*-(4-methoxyphenyl)-1-(1-methyl-1*H* indole-2-yl)ethane-1-imine **9b**. Method A; purified by preparative chromatography using 10% ethyl acetate-hexane mixture as mobile phase. Yellow solid; yield 30%.^{1}H NMR (400 MHz, Chloroform-*d*) *δ* 7.64 (dq, *J* = 7.9, 0.8 Hz, 1H), 7.37 (dq, *J* = 8.4, 0.9 Hz, 1H), 7.30 (ddd, *J* = 8.2, 6.9, 1.1 Hz, 1H), 7.11

(ddt, J = 7.7, 6.9, 0.9 Hz, 1H), 7.00 (d, J = 0.8 Hz, 1H), 6.96 – 6.85 (m, 2H), 6.82 – 6.69 (m, 2H), 4.15 (s, 3H), 3.81 (s, 3H), 2.30 (s, 3H). ^{13}C NMR (176 MHz, $CDCl_3$) δ 160.4, 156.2, 144.4, 140.3, 137.8, 135.1, 126.6, 125.4, 124.1, 121.7, 120.2, 114.70, 111.3, 110.2, 107.5 55.7, 33.1, 18.8. HRMS (EI) m/z Calcd. for $C_{18}H_{19}ON_2$ [M + H]$^+$: 279.1492, found 279.1493.

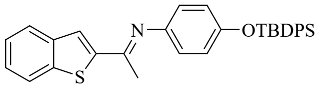

(*E*)-1-(benzo[*b*]thiophen-2-yl)-*N*-(4-((*tert*-butyldiphenylsilyl)oxy)phenyl)ethan-1-imine **10**. Method A; purified by preparative chromatography using 10% ethyl acetate-hexane mixture as mobile phase. Yellow solid; yield: 28%. ^{1}H NMR (400 MHz, $CDCl_3$) δ 7.82 – 7.74 (m, 2H), 7.74 – 7.68 (m, 4H), 7.63 (d, J = 0.9 Hz, 1H), 7.44 – 7.38 (m, 2H), 7.38 – 7.29 (m, 6H), 6.77 – 6.72 (m, 2H), 6.61 – 6.56 (m, 2H), 2.26 (s, 3H), 1.09 (s, 9H).

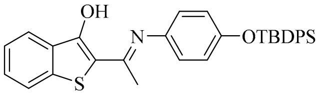

(*E*)-2-(1-(4-((*tert*-butyldiphenylsilyl)oxy)phenyl)imino)ethyl)benzo[b]thiophene-3-ol **11**. Method A; purified by preparative chromatography using 10% ethyl acetate-hexane mixture as mobile phase. Yellow solid; yield: 6%. ^{1}H NMR (700 MHz, Chloroform-*d*) δ 7.96 (dd, J = 7.9, 3.3 Hz, 1H), 7.57 (dd, J = 7.9, 2.8 Hz, 1H), 7.52 (t, J = 7.6 Hz, 1H), 7.48 – 7.43 (m, 1H), 7.40 (q, J = 8.1, 5.5 Hz, 1H), 7.30 – 7.26 (m, 1H), 7.07 – 7.02 (m, 2H), 6.86 – 6.82 (m, 2H), 2.36 – 2.20 (m, 3H), 1.03 – 0.91 (m, 8H), 0.28 – 0.12 (m, 5H).

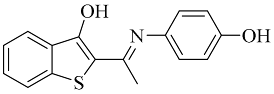

(*E*)-2-(1-((4-hydroxyphenyl)imino)ethyl)benzo[*b*]thiophen-3-ol **12**. Method B; purified by preparative chromatography using 40% ethyl acetate-hexane mixture as mobile phase. Yellow solid; yield: 84%. ^{1}H NMR (700 MHz, MeOH-d_4) δ 7.83 (d, J = 7.8 Hz, 1H), 7.60 (dd, J = 8.0, 2.8 Hz, 1H), 7.45 (t, J = 7.6 Hz, 1H), 7.25 (t, J = 7.0 Hz, 1H), 7.12 – 7.04 (m, 2H), 6.80 (t, J = 5.3 Hz, 2H), 2.29 (d, J = 2.9 Hz, 3H). ^{13}C NMR (176 MHz, $CDCl_3$) δ 182.17, 161.85, 159.57, 154.99, 142.34, 134.80, 131.31, 130.04, 127.03, 125.36,

124.28, 123.59, 116.49, 56.21, 19.48, 14.35. HRMS (ESI) m/z Calcd. for $C_{16}H_{14}O_2NS$ $[M+H]^+$: 284.0740, found 284.0738.

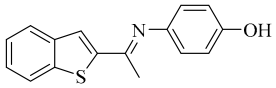

(*E*)-4-((1-(benzo[*b*]thiophen-2-yl)ethylidene)amino)phenol **13**. Method B; purified by preparative chromatography using 30% ethyl acetate-hexane mixture as mobile phase. Yellow solid; yield: 65%. ^{1}H NMR (700 MHz, $CDCl_3$) δ 7.79 (dd, J = 23.2, 7.7 Hz, 2H), 7.65 (s, 1H), 7.34 (dt, J = 18.0, 7.3 Hz, 2H), 6.93 (d, J = 8.0 Hz, 2H), 6.72 (d, J = 8.0 Hz, 2H), 2.32 (s, 3H). ^{13}C NMR (176 MHz, $CDCl_3$) δ 160.91, 153.26, 146.99, 142.83, 141.22, 139.81, 125.95, 125.29, 124.57, 124.43, 122.58, 121.59, 115.82, 63.24, 52.91, 17.15, 8.05. HRMS (ESI) m/z Calcd. for $C_{16}H_{14}ONS$ $[M+H]^+$: 268.0791, found 268.0790.

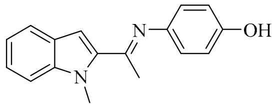

(*E*)-4-((1-(1-methyl-1*H*-indol-2-yl)ethylidene)amino)phenol **14**. Method B; purified by preparative chromatography using 20% ethyl acetate-hexane mixture as mobile phase. Brown solid; yield: 53%. ^{1}H NMR (700 MHz, $CDCl_3$) δ 7.64 (d, J = 7.9 Hz, 1H), 7.37 (d, J = 8.3 Hz, 1H), 7.29 (t, J = 7.7 Hz, 1H), 7.11 (t, J = 7.4 Hz, 1H), 7.00 (s, 1H), 6.83 (d, J = 8.1 Hz, 2H), 6.70 (d, J = 8.1 Hz, 2H), 4.14 (s, 3H), 2.29 (s, 3H). ^{13}C NMR (176 MHz, $CDCl_3$) δ 160.58, 151.93, 144.53, 140.15, 137.82, 126.60, 124.11, 121.74, 121.17, 120.20, 115.90, 110.22, 107.56, 33.16, 18.85. HRMS (ESI) m/z Calcd. for $C_{17}H_{17}ON_2$ $[M+H]^+$: 265.1330, found 265.1335.

Supplementary Figures

Figure S1

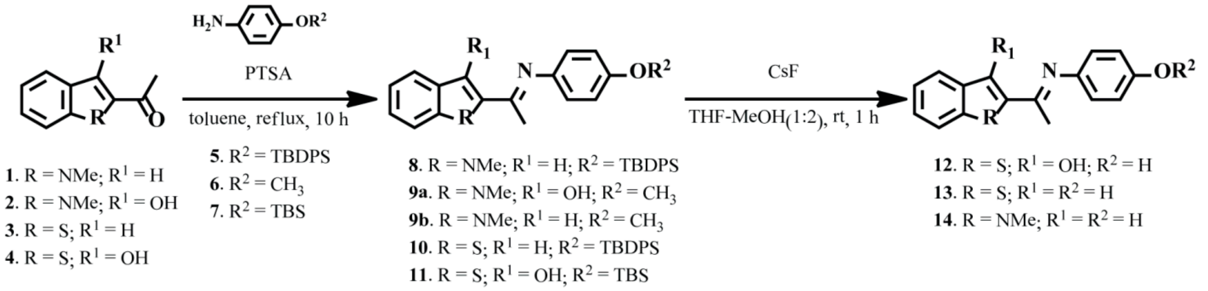

Synthesis of ketimines 9a-b, 12-14

Fig. S1. Synthesis of ketimines 9a-b, 12-14. Ketimines **9**-**11** were synthesized from the corresponding methylketones **1**-**4** and anisidine (**6**) or *O*-silyl-protected *p*-hydroxyaniline (**5** and **7**) using catalytic amount of *p*-toluenesulfonic acid and toluene as solvent. The reactions were performed in Dean-Stark apparatus to remove water. Silyl deprotection of compounds **8**, **10, and 11** were performed by using CsF in methanol-THF mixture to give the final target molecules **12**-**14**.

Figure S2

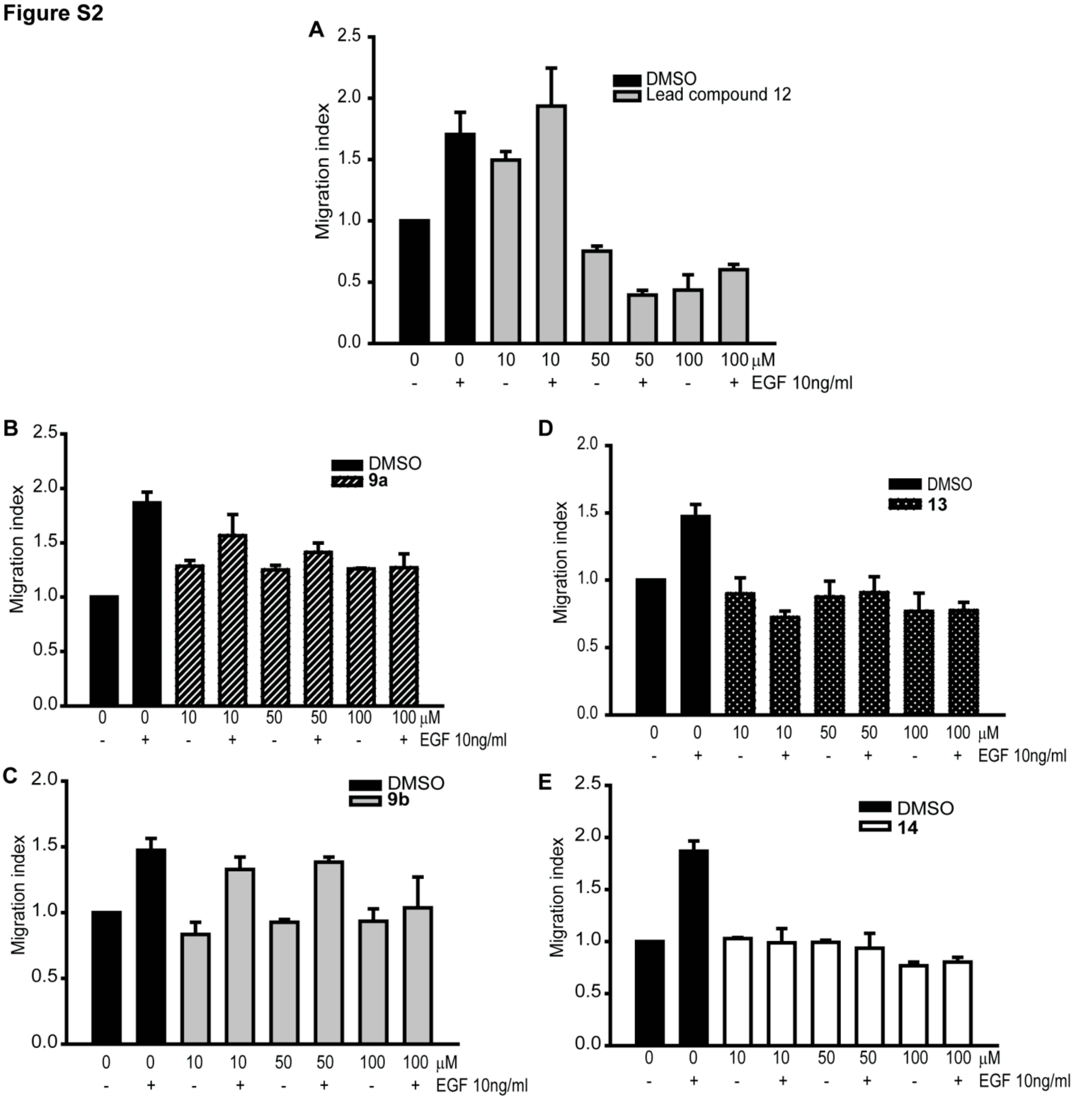

Fig. S2. Optimization of the dosage of the small molecule inhibitors, using PC3 cell lines. A. Transwell migration assay of PC3 cells treated with different concentrations (10, 50, 100 μM) of compound **12**, in presence and absence of EGF treatments (10 ng/ml). Results are expressed as migration index. Each bar represents

mean ± SEM of two different inserts of one experiment. **B.** Transwell migration assay of PC3 cells treated with different concentrations (10, 50, 100 μM) of compound **9a** with or without EGF treatments (10 ng/ml). **C.** Transwell migration assay of PC3 cells treated with different concentrations (10, 50, 100μM) of compound **9b** with or without EGF treatments (10 ng/ml). **D.** Transwell migration assay of PC3 cells treated with different concentrations (10, 50, 100μM) of compound **13** with or without EGF treatments (10ng/ml). **E.** Transwell migration assay of PC3 cells treated with different concentrations (10, 50, 100 μM) of compound **14** with or without EGF treatments (10ng/ml).

Figure S3

11/2/2017 https://www.ebi.ac.uk/Tools/services/rest/clustalo/result/clustalo-I20171102-160306-0065-89360785-pg/aln-clustal

```
CLUSTAL O(1.2.4) multiple sequence alignment


GNAI1      MGCTLSAEDKAAVERSKMIDRNLREDGEKAAREVKLLLLGAGESGKSTIVKQMKIIHEAG
GNAI2      MGCTVSAEDKAAAERSKMIDKNLREDGEKAAREVKLLLLGAGESGKSTIVKQMKIIHEDG
           ****:*******.*******:************************************* *

GNAI1      YSEEECKQYKAVVYSNTIQSIIAIIRAMGRLKIDFGDSARADDARQLFVLAGA-AEEGFM
GNAI2      YSEEECRQYRAVVYSNTIQSIMAIVKAMGNLQIDFADPSRADDARQLFALSCTAEEQGVL
           ******:**:***********:**::***.*:***.* :*********.*: :  *:*.:

GNAI1      TAELAGVIKRLWKDSGVQACFNRSREYQLNDSAAYYLNDLDRIAQPNYIPTQQDVLRTRV
GNAI2      PDDLSGVIRRLWADHGVQACFGRSREYQLNDSAAYYLNDLERIAQSDYIPTQQDVLRTRV
             :*:***:*** * ******.******************:****  :*************

GNAI1      KTTGIVETHFTFKDLHFKMFDVGGQRSERKKWIHCFEGVTAIIFCVALSDYDLVLAEDEE
GNAI2      KTTGIVETHFTFKDLHFKMFDVGGQRSERKKWIHCFEGVTAIIFCVALSAYDLVLAEDEE
           ************************************************* **********

GNAI1      MNRMHESMKLFDSICNNKWFTDTSIILFLNKKDLFEEKIKKSPLTICYPEYAGSNTYEEA
GNAI2      MNRMHESMKLFDSICNNKWFTDTSIILFLNKKDLFEEKITHSPLTICFPEYTGANKYDEA
           ***************************************.:******:***:*:*.*:**

GNAI1      AAYIQCQFEDLNKRKDTKEIYTHFTCATDTKNVQFVFDAVTDVIIKNNLKDCGLF
GNAI2      ASYIQSKFEDLNKRKDTKEIYTHFTCATDTKNVQFVFDAVTDVIIKNNLKDCGLF
           *:***.:************************************************
```

Fig. S3. Alignment of $G\alpha_i1$ and $G\alpha_i2$ proteins, using Clustal Omega. Multiple sequence alignment of $G\alpha_i1$ and $G\alpha_i2$ amino acidic sequences reveals that the GTP-binding sites (yellow) are conserved in both the proteins.

Figure S4

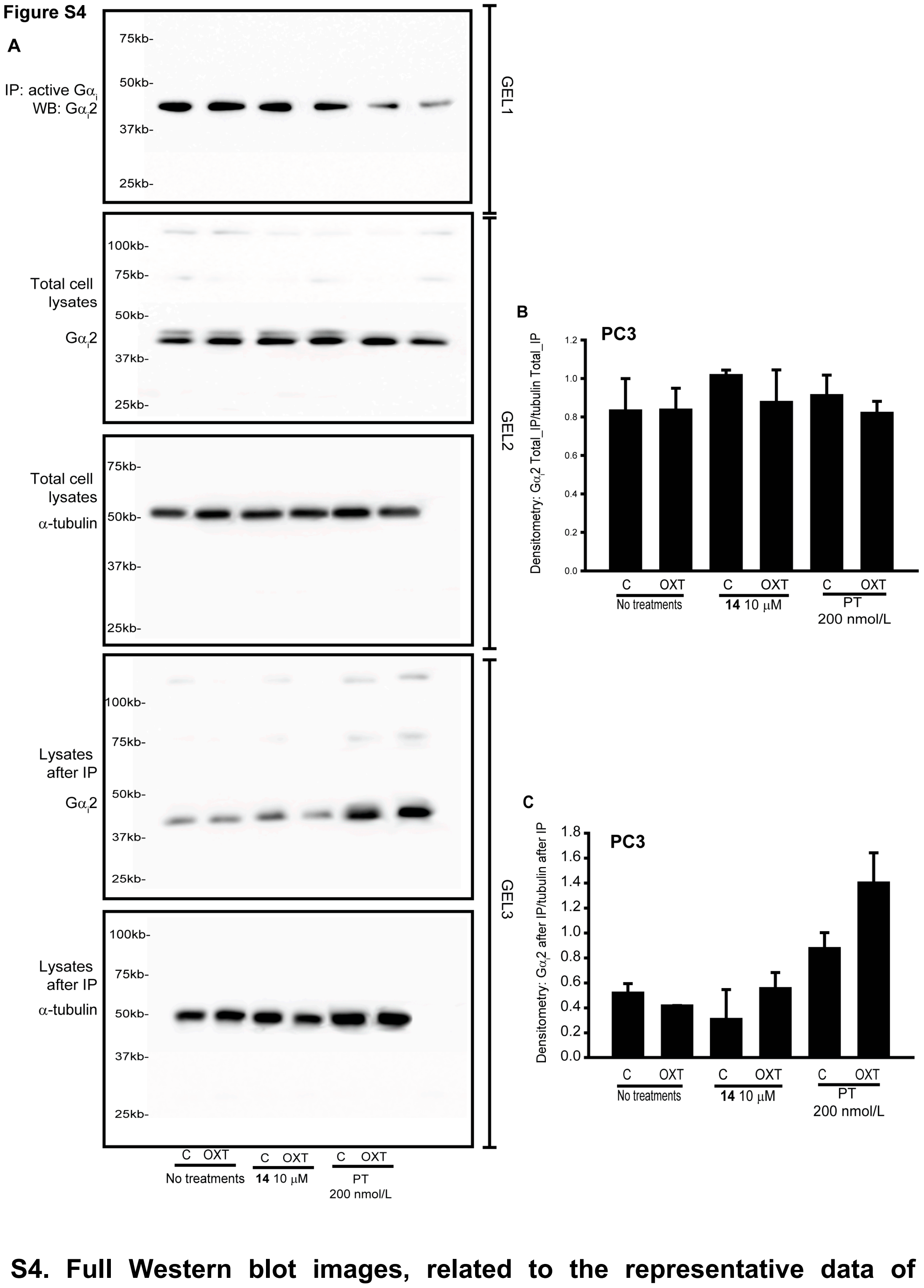

Fig. S4. Full Western blot images, related to the representative data of the Immunoprecipitation experiments. A. Full blots of the Immunoprecipitation experiment. The densitometry of the first panel, that is the IP of active Gα_i and western

blot for $G\alpha_i2$ experiment is presented in Fig. 4B (right panel), as average of three different experiments ($G\alpha_i2$ IP/$G\alpha_i2$ Total Cell Lysate), in the main manuscript. **B.** Densitometric analysis of $G\alpha_i2$ protein expression, using total cell lysates, was performed. **C.** Densitometric analysis of $G\alpha_i2$ protein expression, using lysates after IP, was performed. In all the experiments the molecular weight markers are not coming up because the $G\alpha_i2$ antibody (AbCam) used is very specific and it does not interfere with it.

Figure S5

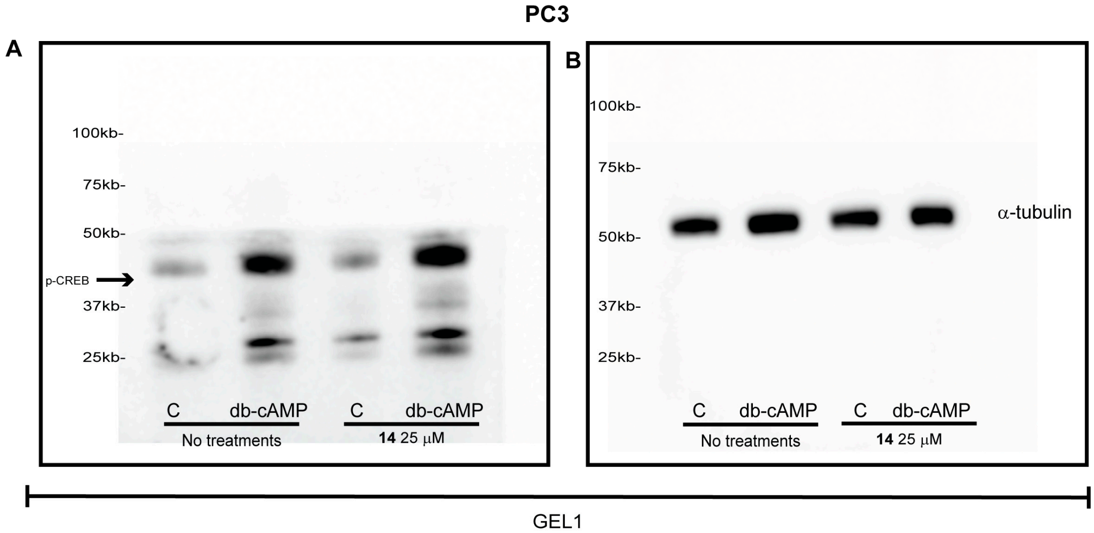

Fig. S5. Full Western blot images, related to the representative data of the levels of p-CREB (A) and α-tubulin (B), after treatments with compound 14, in presence of db-cAMP. The densitometric analysis of this experiment is presented in Fig.4A in the main manuscript. In these experiments the molecular weight markers are not coming up because the p-CREB antibody used is very specific and it does not interfere with it.

Figure S6

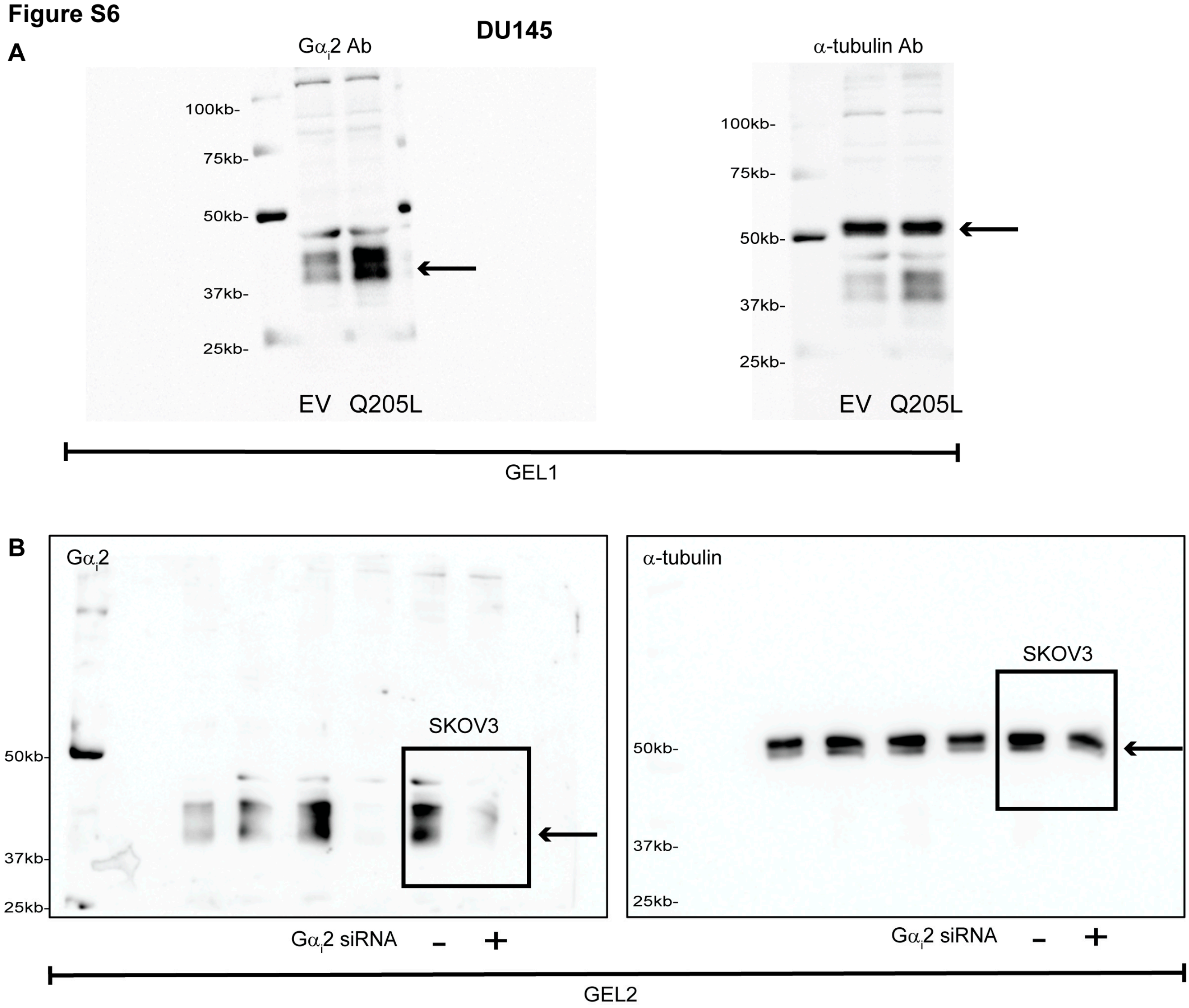

Fig. S6. Full Western blot images, related to the representative data of the overexpression and knockdown of Gα$_i$2 protein experiments in DU145 (Gα$_i$2-Q205L), and SKOV3. A. DU145 cell lines were transiently transfected with EV and Gα$_i$2-

Q205L plasmids and used for further assays. Western blot analysis was performed to confirm the overexpression. **B.** SKOV3 cells were transiently transfected with Control and $G\alpha_i2$ siRNAs and used for further assays. Western blot analysis was performed to confirm the knockdown.

Figure S7

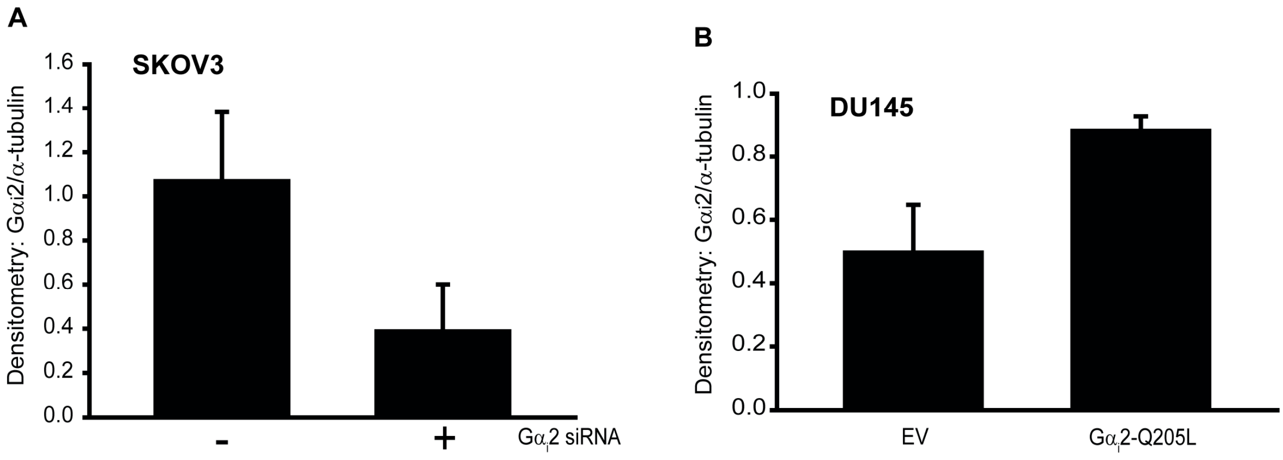

Fig. S7. Densitometric analysis of Western blot images: A. Densitometric analysis of SKOV3 cells, transfected with Control and Gα$_i$2 siRNAs, was performed. **B.** Densitometric analysis of DU145 overexpressing EV and Gα$_i$2-Q205L was performed.

References

1. Malkov, A.V.; Vrankova, K.; Stoncius, S.; Kocovsky, P. Asymmetric reduction of imines with trichlorosilane, catalyzed by sigamide, an amino acid-derived formamide: scope and limitations. *J Org Chem* 2009, *74*, 5839-5849, doi:10.1021/jo900561h.

2. Xie, Y.Q.; Huang, Z.L.; Yan, H.D.; Li, J.; Ye, L.Y.; Che, L.M.; Tu, S. Design, synthesis, and biological activity of oxime ether strobilurin derivatives containing indole moiety as novel fungicide. *Chem Biol Drug Des* 2015, *85*, 743-755, doi:10.1111/cbdd.12460.

3. Unangst, P.C.; Brown, R.E.; Fabian, A.; Fontseré, F. 2 - Indolyl ketone synthesis. *Journal of Heterocyclic Chemistry* 1979, *16*, 661-666.

4. Handayani, M.; Gohda, S.; Tanaka, D.; Ogawa, T. Design and synthesis of perpendicularly connected metal porphyrin-imide dyads for two-terminal wired single molecular diodes. *Chemistry* 2014, *20*, 7655-7664, doi:10.1002/chem.201402052.

5. Cheng, P.; Zhou, J.; Qing, Z.; Kang, W.; Liu, S.; Liu, W.; Xie, H.; Zeng, J. Synthesis of 5-methyl phenanthridium derivatives: a new class of human DOPA decarboxylase inhibitors. *Bioorg Med Chem Lett* 2014, *24*, 2712-2716, doi:10.1016/j.bmcl.2014.04.047.
